# Gamma‐glutamyl transferase: A potential biomarker for pancreas steatosis in patients with concurrent obesity, insulin resistance and metabolic dysfunction‐associated steatotic liver disease

**DOI:** 10.1111/cob.12712

**Published:** 2024-10-22

**Authors:** Chileka Chiyanika, Elizabeth Shumbayawonda, Michele Pansini, Kin Hung Liu, Terry Cheuk‐Fung Yip, Vincent Wai‐Sun Wong, Winnie Chiu Wing Chu

**Affiliations:** ^1^ Department of Health Technology and Informatics The Hong Kong Polytechnic University Hong Kong China; ^2^ Department of Imaging and Interventional Radiology, Prince of Wales Hospital The Chinese University of Hong Kong Hong Kong China; ^3^ Translational Science Perspectum Diagnostic limited Oxford UK; ^4^ Clinica Di Radiologia EOC, Istituto Di Imaging Della Svizzera Italiana (IIMSI) Ente Ospedaliero Cantonale Lugano Switzerland; ^5^ John Radcliffe Hospital Oxford University Hospitals NHS Foundation Trust Oxford UK; ^6^ Medical Data Analytic Centre The Chinese University of Hong Kong Hong Kong China; ^7^ Department of Medicine and Therapeutics The Chinese University of Hong Kong Hong Kong China; ^8^ Institute of Digestive Disease The Chinese University of Hong Kong Hong Kong China

**Keywords:** fatty pancreas, gamma‐glutamyl transferase, insulin resistance, metabolic dysfunction‐associated steatotic liver disease, metabolic syndrome, obesity

## Abstract

To evaluate the relationship between serum gamma‐glutamyl transferase (GGT) levels and fatty pancreas in subjects with concurrent obesity, insulin resistance and metabolic dysfunction‐associated steatotic liver disease (MASLD) without a history of pancreatitis. From March 2019 to September 2021, 31 adult subjects with concurrent obesity and MASLD were recruited as part of the study investigating the biological impact of bariatric surgery and lifestyle modification on obesity. Chemical shift encoded MRI of the abdomen, LiverMultiScan, anthropometric, clinical and blood biochemistry analyses were performed prior to any intervention at baseline. GGT (*p* <.001) was significantly different between those ‘with fatty pancreas’ and ‘without fatty pancreas’ groups. GGT (*p* <.001) was significantly different between those ‘with both metabolic syndrome and fatty pancreas’ and those ‘with metabolic syndrome but without fatty pancreas.’ GGT (*p* <.001) was also significantly different between those ‘with both diabetes and fatty pancreas’ and those ‘with diabetes but without fatty pancreas’. Logistic regression analysis showed that abnormal GGT levels (*p* = .010) and Hypertension (*p* = .045) were significant independent predictors of fatty pancreas. GGT was associated with fatty pancreas by an odds ratio 7.333 (95% [CI]: 1.467–36.664), while the AUROC of GGT in determining fatty pancreas was 0.849. Elevation in serum GGT might be a potential marker to identify fatty pancreas.

**What is already known about this subject?**

- Fatty pancreas and Metabolic dysfunction‐associated steatotic liver disease are common manifestations of obesity.
- Fatty pancreas can progress to pancreatic cancer.
- Elevated serum enzyme gamma‐glutamyl transferase (GGT) is commonly associated with liver damage.

**What this study adds?**

- Fatty pancreas may be associated with elevated GGT, irrespective of metabolic syndrome, diabetes, hypoalphalipoproteinemia, MASH, and medication known to increase GGT levels.
- When serum GGT is elevated, the likelihood of having fatty pancreas could be 7.3 times higher.
- Abnormal GGT levels and hypertension were found to be significant predictors of fatty pancreas, whereas the diagnostic accuracy (AUC) of GGT for fatty pancreas was found to be 0.849.

## 1 INTRODUCTION

Fatty pancreas is one of the conditions associated with obesity, with a global prevalence ranging from 44% to 58% among the population with obesity.^1^, ^2^ It can progress from simple steatosis to acute non‐alcoholic steatopancreatitis, chronic fibrosing non‐alcoholic steatopancreatitis, and pancreatic cancer.^3^, ^4^ Pancreatic fat accumulation is a significant predictor of beta‐cell dysfunction, insulin resistance, and metabolic syndrome.^5^, ^6^ Recently, Chan et al.^7^ demonstrated that fatty pancreas is independently associated with subsequent development of T2DM at 10 years.

The enzyme gamma‐glutamyl transferase (GGT) is found in serum and cell membrane surfaces of the kidneys, bile duct, pancreas, gall bladder, spleen, heart, brain, and seminal vesicles.^8^ It plays a role in maintaining intracellular homeostasis from oxidative stress.^9^ Elevated serum GGT is commonly associated with liver damage,^10^ but recent studies have linked it to cardiovascular disease,^11^ metabolic syndrome,^12^ coronary artery disease,^11^ chronic kidney disease,^13^ T2DM,^14^ and hip fractures.^15^

In relation to the pancreas, serum GGT elevation is linked to pancreatic cancer,^16^ but data on the relationship between fatty pancreas is scarce. Given the clinical implications of fatty pancreas, there is a need for early markers of this condition, to facilitate comprehensive patient assessment and institution of early intervention in a cost‐effective manner. Thus, as GGT is a simple, quick, and cheap routine test in clinical settings in comparison to MRI (gold standard to quantify pancreatic fat), the aim of this study was to evaluate the relationship between serum GGT levels and fatty pancreas in subjects with obesity and with both insulin resistance and metabolic dysfunction‐associated steatotic liver disease (MASLD) without a history of pancreatitis.

## 2 MATERIALS AND METHODS

### 2.1 Study design and participants

This cross‐sectional study was part of the prospective study investigating the biological impact of bariatric surgery and lifestyle modification on Chinese subjects with obesity. However, this current study only analysed the baseline data before the subjects commenced the intervention, and therefore the interventions had no effect on this study. Between March 2019 to September 2021, 31 subjects were recruited to participate in this study after fulfilling the selection criteria. Our institutional review board approved the study (reference number 2018.612) and written informed consent was obtained from all the participants.

### 2.2 Selection criteria

The study included subjects of Chinese ethnicity aged 18–65 years, BMI ≥27.5 kg/m^2^ (adjusted criteria for Asian population^17^), with a diagnosis of MASLD based on MRI PDFF ≥5.5%, low (to no) alcohol consumption (<30 g/day for men and <20 g/day for women), and any one of the following metabolic factors as defined by Rinella et al.^18^: (a) waist circumference ≥90 cm in Asian men and ≥80 cm in Asian women‐ethnically adjusted, (b) fasting serum glucose ≥5.6 mmol/L (100 mg/dL) or 2‐h post‐load glucose levels ≥7.8 mmol/L (≥140 mg/dL) or HbA1c ≥5.7% (39 mmol/L) or type 2 diabetes or treatment for type 2 diabetes (c) blood pressure >130/85 mmHg or specific antihypertensive drug treatment, (d) plasma triglycerides ≥1.70 mmol/L (150 mg/dL) or lipid‐lowering treatment, and (e) plasma HDL‐cholesterol ≤1.0 mmol/L (40 mg/dL) in men and ≤1.3 mmol/L (50 mg/dL) in women or lipid‐lowering treatment. Exclusion criteria included: any contraindications to MRI, other kind of hepatic diseases, or under medications known to affect liver fat accumulation and with a history of pancreatitis.

### 2.3 Clinical assessment and anthropometric measurements

Anthropometric measurements including body weight, height, waist circumference, and diastolic and systolic blood pressure were recorded. BMI was calculated as weight in kilograms (kg) divided by height in metre squared (m^2^). BMI was then used to categorise obesity status of the subjects using the World Health Organization region‐specific classification of weight.^17^ Blood tests including liver enzymes, glucose, and lipids were conducted after 8 hours of fasting, and within 7 days from MRI examination. The subjects' comprehensive past medical history, drug history/current use of medications, smoking history, and alcohol consumption were recorded.

### 2.4 Insulin resistance/type 2 diabetes mellitus

Insulin resistance was estimated using the homeostasis model assessment‐insulin resistance (HOMA‐IR), calculated as HOMA‐IR = fasting plasma glucose (mmol/L) × insulin (mIU/L)/22.5.^19^ Homeostasis model assessment beta‐cell function (HOMA‐B), calculated as HOMA‐B = [20 × Fasting insulin (mIU/L)]/[glucose (mmol/L) − 3.5]^20^ was used to estimate beta‐cell function. Insulin resistance was defined as HOMA‐IR ≥1.4 in non‐diabetic subjects and ≥2.0 in diabetic subjects.^21^ The diagnosis of T2DM was based on the criteria set by World Health Organization.^22^

### 2.5 Metabolic syndrome and abnormal GGT

Metabolic syndrome was defined using the harmonised criteria,^23^ that is, the presence of at least any three out of the five: central obesity, hyperglycaemia, hypertension, dyslipidaemia, hypoalphalipoproteinemia, and/or receiving treatment for any of the above metabolic abnormalities. Whereas normal GGT range was 8–38 IU/L and cut‐off for normal was set at <40 IU/L.^24^ For uniformity, this GGT level cut off in both women and men was used in this study as there is a wide variation in reported thresholds for abnormal GGT levels among laboratories as well as reported in literature.

### 2.6 MRI data acquisition and analysis

All 31 subjects underwent MRI examination using Philips Achieva 3.0 T MRI Scanner (Philips Medical System, Best, The Netherlands) equipped with a 16‐channel SENSE‐XL‐Torso array coil. The subjects had to fast for at least 8 h before the examination. Chemical‐shift water‐fat images were acquired by a 3D spoiled multi‐echo mDIXON sequence to yield co‐registered water, fat, fat‐fraction, and T2* image series of the whole abdomen. LiverMultiScan was also performed using an ECG‐triggered Shortened Modified Look‐Locker Inversion sequence to obtain iron corrected liver T1 values.

### 2.7 Quantification of liver PDFF

Nine elliptical regions of interest (ROIs) set to 4 cm^2^ (as described by Campo et al.^25^) were placed into all nine Couinaud liver segments localised on PDFF maps avoiding the hepatic blood vessels, bile ducts, and motion artefacts using the Philips DICOM Viewer software version R3.0‐SP15 (Philips Healthcare, Netherlands). The median liver PDFF from all the nine segments was used for analysis.

### 2.8 Quantification of pancreas PDFF and T2*

Pancreas PDFF and T2* obtained at the same point were measured using the fat fraction and T2* image series, respectively. Three ROIs set to 1 cm^2^ were drawn on the head, body, and tail of the pancreas thrice in any slice showing the pancreas clearly (avoiding the pancreatic duct and splenic vein) using the same viewer software. The median PDFF/T2* from the three ROIs were averaged to get the median pancreatic fat fraction/T2* values. The definition of fatty pancreas was based on pancreas PDFF ≥6.2%.^26^ In our similar study, the interclass correlation coefficient absolute agreement of all the above measurements ranged from of 0.860 to 0.908.^5^

### 2.9 Iron corrected liver T1/T2* calculation

Liver MR data were post‐processed to characterise disease activity using iron corrected liver T1 (cT1) and T2* using Liver*MultiScan®* (Oxford, UK).^27^ Briefly, the algorithm eliminates the biases introduced by excess iron as determined by T2* values to enable T1 measurements that are iron‐corrected. Details of this algorithm are described by Banerjee et al.^28^ and Tunnicliffe et al.^29^

### 2.10 Quantification of abdominal white adipose tissue

Abdominal white adipose tissue was separated into subcutaneous and visceral adipose tissues (SAT and VAT) using a validated in‐house algorithm.^30^ Briefly, this algorithm detects and removes the narrow connecting regions between SAT and VAT using a spoke‐like template constructed by Bresenham's Line and Midpoint Circle method, which was applied over the adipose tissue to automatically separate SAT and VAT.

### 2.11 Statistical analysis

To ensure the reliability and validity of the study results, we performed a power analysis. We first calculated the Cohen's d using the mean and standard deviation of GGT for those with and without fatty pancreas, yielding an effect size of 0.882301. We then performed a two‐tailed analysis with this effect size, setting the significance level (alpha) at 0.05 and using the known sample sizes [without fatty pancreas = 15 and with fatty pancreas = 16] and *β*/*α* ratio (0.94), which allowed us to achieve a statistical power (1 − err prob) of 0.84. Therefore, with an effect size of 0.882301 and a statistical power of 84%, indicating a very low likelihood of a Type II error, the results presented are both statistically and practically significant.

Data were expressed as median (range) unless stated otherwise. Mann–Whitney and Fisher's exact tests were used to compare groups accordingly. Area under receiver operating characteristic (AUROC) curve was used to test the diagnostic performance of test variables. Odds ratio (OR) was performed to determine relative risks. Logistic regression analysis was performed to determine independent predicting variables with correction of other variables. Pearson's correlation coefficient was used to test the relationship among variables. All tests were two‐sided and *p*‐values <.05 were considered statistically significant. Statistical analyses were performed by using the SPSS software, version 28.0 (IBM, Chicago, IL).

## 3 RESULTS

### 3.1 Patient characteristics

#### 3.1.1 Clinical and anthropometrics

A total of 31 participants (71% female, median age 44 years; BMI 38 kg/m^2^) were included in this study. Of these, 16 (52%) had fatty pancreas, all (100%) had fatty liver, all (100%) had insulin resistance, 24 (77.4%) had established T2DM, and 26 (83.9%) had metabolic syndrome. Of this cohort, 12 (52.2%) were on lipid‐lowering drugs, 17 (73.9%) on antidiabetics, while 13 (56.5%) were on antihypertensives. None (0%) had a history of COVID‐19 infection, 13 (42%) had COVID‐19 vaccination while 23 (74%) were on medication known to increase GGT levels within 6 months from MRI examination. Details are shown in Table 1 and supplementary Table S1.

**TABLE 1:** Clinical, anthropometric, blood biochemistry, and imaging characteristics of male and female subjects.

Characteristics	All subjects (*n* = 31)	Males (*n* = 9)	Females (*n* = 22)	*p*‐value
Age (year)	44 (28–52)	44 (36–52)	45 (28–52)	.458
Smoking yes, *n* (%)	6 (19.4)	4 (44)	2 (10)	.768
Drinking yes, *n* (%)	6 (19.4)	2 (22.2)	4 (18.2)	.799
COVID‐19 infection, *n* (%)	0 (0)	0 (0)	0 (0)	1.000
COVID‐19 vaccination, *n* (%)	13 (42)	4 (80)	9 (41)	.727
Medication, *n* (%)	23 (74)	8 (89)	15 (68)	.939
BMI (kg/m^2^)	38 (28.1–44.6)	36 (28.3–42.1)	38 (28.1–44.6)	.572
Waist circumference (cm)	114 (95–127)	114 (95–124)	114 (97–127)	.777
Systolic blood pressure (mm Hg)	126 (100–143)	129 (102–143)	125 (100–134)	.228
Diastolic blood pressure (mm Hg)	80 (57–138)	83 (59–103)	80 (63–138)	.983
Antihypertensive drugs, *n* (%)	13 (56.5)	6 (66.7)	7 (31.8)	.516
Lipid‐lowering drugs, *n* (%)	12 (52.2)	5 (55.6)	7 (31.8)	.799
Insulin resistance, *n* (%)	31 (100)	9 (100)	22 (100)	1.000
T2DM, *n* (%)	24 (77.4)	7 (77.8)	17 (77.3)	.976
Antidiabetic drug, *n* (%)	17 (73.9)	7 (77.8)	10 (45.5)	.741
Metabolic syndrome, *n* (%)	26 (83.9)	8 (88.9)	18 (81.8)	.633
Total cholesterol (mmol/L)	4.2 (0.6–6.2)	4.3 (2.8–6.1)	4.2 (0.6–6.2)	.777
Triglycerides (mmol/L)	1.6 (0.8–5.0)	1.5 (1.2–5.0)	1.6 (0.8–3.1)	.555
HDL‐c (mmol/L)	1.1 (0.6–1.7)	1.0 (0.8–1.5)	1.2 (0.6–1.7)	.071
LDL‐c (mmol/L)	2.4 (1.2–4.5)	2.4 (1.3–4.5)	2.2 (1.2–4.1)	.965
Albumin (mmol/L)	39.5 (5–44)	39 (34–41)	40 (5.0–44.0)	.422
HBA1c (%)	6.7 (5.5–11.7)	6.8 (5.8–11.7)	6.4 (5.5–11.6)	.248
Fasting plasma glucose (mmol/L)	6.4 (3.9–10.9)	6.6 (3.9–10.9)	5.9 (4.4–9.2)	.257
Plasma fasting Insulin (mIU/L)	27.4 (12.8–77.0)	26.4 (12.8–77.0)	27.7 (13.4–42.9)	.874
HOMA‐IR	7.30 (4.22–29.81)	9.01 (4.49–29.81)	7.09 (4.22–14.35)	.572
HOMA‐B	200 (52.70–1320)	187 (52.70–1320)	206 (70.53–543.64)	.700
ALP (IU/L)	63 (40–161)	62 (54–86)	65 (40–161)	.981
ALT (IU/L)	39 (13–132)	39 (13–68)	40 (18–132)	.828
AST (IU/L)	29 (5.2–96.0)	29 (19–37)	32 (16–96)	.267
GGT (IU/L)	32 (2.8–65)	32 (2.8–58)	32 (2.8–65)	.964
VAT (litres)	4.15 (2.77–6.67)	4.68 (3.04–6.67)	4.06 (3.06–5.58)	.200
SAT (litres)	23.3 (12.12–0.04)	19.8 (12.12–33.96)	26.1 (16.38–40.04)	**.024**
VAT/SAT ratio	0.18 (0.10–0.55)	0.23 (0.16–0.55)	0.16 (0.10–0.27)	**.006**
Pancreatic PDFF (%)	6.45 (2.7–23.6)	7.73 (3.6–23.6)	5.63 (2.7–20.9)	.164
Pancreatic T2* (ms)	39.94 (24.33–52.39)	36.7 (34.64–44.31)	40.9 (24.33–52.39)	.182
Liver PDFF (%)	13.89 (6.6–32.5)	11 (6.6–28.7)	17 (10.4–32.5)	.054
Fatty liver, *n* (%)	31 (100)	9 (100)	22 (100)	1.000
Liver cT1 (ms)	890 (693–1062)	788 (693–995)	905 (786–1062)	**.031**
Liver T2* (ms)	19.9 (12.0–29.3)	18 (12.0–24.3)	21 (13–29.3)	**.047**

*Note*: Fischer exact test was used for categorical variables and Mann–Whitney test was used for continuous variables. Medication = use of medication known to increase GGT levels within 6 months from MRI examination. COVID‐19 vaccination/infection = within 6 months from MRI examination. Bold indicates statistically significant values *p* < 0.05.

Abbreviations: ALP, alkaline phosphatase; ALT, alanine aminotransferase; AST, aspartate transaminase; BMI, body mass index; cT1, iron corrected T1; GGT, gamma‐glutamyl transferase; HbA1c, glycosylated haemoglobin; HDL, high density lipoprotein; HOMA‐IR, homeostasis model assessment‐insulin resistance; HOMA‐B, homeostasis model assessment beta; LDL, low density lipoprotein; PDFF, proton density fat fraction, SAT, subcutaneous adipose tissue; T2*, relaxation time, T2DM, type 2 diabetes mellitus.; VAT, visceral adipose tissue.

#### 3.1.2 Blood biochemistry and imaging characteristics

Only VAT/SAT ratio (0.23 vs. 0.16, *p* = .006), SAT (19.8 vs. 26.1 L, *p* = 0.024), cT1 (788 vs. 905 ms, *p* = .031), and liver T2* (18 vs. 21 ms, *p* = .046) were significantly different between males and females, respectively as shown in Table 1.

### 3.2 Group comparison of those with versus without fatty pancreas

The study cohort was then divided into two groups, that is, ‘with fatty pancreas’ and ‘without fatty pancreas’. There was a significant difference in the pancreatic fat content between the groups (8.95 vs. 3.94%, *p* <.001) as well as in the pancreatic T2* (37.15 vs. 41.51 ms, *p* = .026). Also, it was shown that BMI (38.3 vs. 34.6 kg/m^2^, *p* = .030), waist circumference (115 vs. 110 cm, *p* = .029), total cholesterol (4.4 vs. 3.6 mmol/L, *p* = .019), low‐density lipoprotein‐ cholesterol (2.5 vs. 1.9 mmol/L, *p* = .036), AST (26 vs. 32 IU/L, *p* = .043), GGT (48 vs. 25 IU/L, *p* < .001) and liver T2* (18.6 vs. 21.3 ms, *p* = .030) were significantly different between the ‘with fatty pancreas’ and ‘without fatty pancreas’ groups, respectively (Table 2). Besides, using a cut‐off point of GGT at ≥40 IU/L for abnormal GGT levels, only 3 (20%) in the ‘without fatty pancreas group’ had abnormal GGT levels, whereas 11 (65%) in the ‘with fatty pancreas group’ had abnormal GGT levels and the difference between groups was significantly different (*p* = .030).

**TABLE 2:** Clinical and laboratory characteristics of subjects with and without fatty pancreas.

Characteristics	Without fatty pancreas (*n* = 15)	With fatty pancreas (*n* = 16)	*p*‐value
Age (year)	47 (35–52)	40 (28–52)	.054
Male/female *n* (%)	3/12 (20/80)	6/10 (37.5/62.5)	.433
Smoking yes/no, *n* (%)	1/14 (7/93)	3/13 (18/82)	1.000
Drinking yes/no, *n* (%)	3/12 (20/80)	3/13 (18.8/81.2)	1.000
Covid‐19 infection, *n* (%)	0 (0)	0 (0)	1.000
Covid‐19 vaccination, *n* (%)	8 (53)	5 (31)	.176
Medication, *n* (%)	12 (80)	11 (69)	.345
BMI (kg/m^2^)	34.6 (28.3–41.3)	38.3 (33.6–44.6)	**.030**
Waist circumference (cm)	110 (95–121)	115 (110–127)	**.029**
Systolic blood pressure (mm Hg)	125 (100–142)	129 (107–143)	.192
Diastolic blood pressure (mm Hg)	80 (67–138)	75 (59–103)	.332
Antihypertensive drugs, *n* (%)	9 (75)	4 (36.4)	.068
Total cholesterol (mmol/L)	3.6 (0.6–6.2)	4.4 (3.3–6.1)	**.019**
Triglycerides (mmol/L)	1.6 (0.9–1.7)	1.5 (0.8–5.0)	.513
HDL‐c (mmol/L)	1.1 (0.6–1.7)	1.2 (0.8–1.5)	.719
LDL‐c (mmol/L)	1.9 (1.2–4.1)	2.5 (1.5–4.5)	**.036**
Albumin (mmol/L)	40 (5.0–44.0)	39 (34–43)	.402
Lipid‐lowering drugs, *n* (%)	9 (69.3)	6 (70.6)	.546
HBA1c (%)	6.5 (5.5–8.4)	6.9 (5.5–11.7)	.692
Fasting plasma glucose (mmol/L)	6.4 (3.9–9.1)	6.3 (5.1–10.9)	.440
Plasma fasting Insulin (mIU/L)	28.2 (17.1–36.7)	25.5 (12.8–77.0)	.633
HOMA‐IR	6.52 (4.22–14.35)	7.70 (4.35–29.81)	.884
HOMA‐B	192.1 (82.33–1320)	201 (52.7–616)	.603
Insulin resistance, *n* (%)	15 (100)	16 (100)	.000
T2DM, *n* (%)	12 (80)	12 (75)	.000
Antidiabetic drug, *n* (%)	9 (75)	8 (72.7)	.000
Metabolic syndrome, *n* (%)	14 (93.3)	14 (82.4)	1.000
ALP (IU/L)	53 (32–161)	76 (50–102)	.095
ALT (IU/L)	40 (18–132)	38.5 (13–107)	.782
AST (IU/L)	32 (19–96)	26 (16–45)	**.043**
GGT (IU/L)	25 (2.8–53)	48 (30–65)	**<.001**
Abnormal GGT, *n* (%)	3 (20)	11 (65)	**.030**
VAT (litres)	4.14 (3.04–6.67)	4.25 (3.06–5.88)	.352
SAT (litres)	23.73 (12.12–40.04)	26.34 (16.10–33.96)	**.045**
VAT/SAT ratio	0.18 (0.10–0.55)	0.16 (0.11–0.24)	.642
Pancreatic PDFF (%)	3.94 (2.7–5.8)	8.95 (6.4–23.6)	**<.001**
Pancreatic T2* (ms)	41.51 (28.65–51.23)	37.15 (24.33–45.68)	**.026**
Liver PDFF (%)	12.77 (5.6–26.3)	15.33 (7.8–32.5)	.707
Fatty liver, *n* (%)	15 (100)	16 (100)	1.000
Liver cT1 (ms)	924 (724–1062)	891 (780–995)	.441
MASH, *n* (%)	5 (33)	5 (29)	.852
Liver T2* (ms)	21.3 (14.6–29.3)	18.6 (12.0–23.9)	**.030**

*Note*: Mann–Whitney test done for continuous variables and Fischer exact test for categorical variables. MASH was determined by setting cT1 at 925 ms. Bold indicates statistically significant values *p* < 0.05.

Abbreviations: ALP, alkaline phosphatase; ALT, alanine aminotransferase; AST, aspartate transaminase; BMI, body mass index; cT1, iron corrected T1; GGT, gamma‐glutamyl transferase; HbA1c, glycosylated haemoglobin; HOMA‐IR, homeostasis model assessment‐insulin resistance; HOMA‐B, homeostasis model assessment beta; HDL, high density lipoprotein; LDL, low density lipoprotein; MASH, metabolic dysfunction‐associated steatohepatitis; PDFF, proton density fat fraction; SAT, subcutaneous adipose tissue; T2*, relaxation time; VAT, visceral adipose tissue.

The cT1 values between subjects with and without fatty pancreas were not significantly different (891 ms vs. 924 ms, *p* = .441), nor was the liver fat content (15.33% vs. 12.77%, *p* = .707), as shown in Table 2. Using cT1 as a biomarker to diagnose metabolic dysfunction‐associated steatohepatitis (MASH) with a cutoff point of 925 ms, as indicated in previous studies,^31^, ^32^ 5 subjects (33%) without fatty pancreas had MASH, while 5 subjects (29%) with fatty pancreas had MASH (*p* = .852). The cT1 values between these two groups [with fatty pancreas and with MASH, cT1 = 975 ms (937–995 ms) and without fatty pancreas but with MASH‐cT1 = 954 ms (940–1062 ms)] were not significantly different (*p* = .917). However, the difference in GGT levels between these two groups were statistically significant [with fatty pancreas and with MASH, GGT levels = 58 (48–61 IU/L) vs. without fatty pancreas but with MASH, GGT levels = 32 (2.8–53 IU/L), *p* = .028]. This indicates that the presence of MASH did not influence the GGT levels. Similarly, the non‐significant difference in liver fat content between the two groups suggests that liver fat or steatosis grade did not influence the GGT levels.

### 3.3 Metabolic syndrome and fatty pancreas

Given the close relationship between metabolic syndrome and GGT, we further divided the cohort into those ‘with both metabolic syndrome and fatty pancreas’ and those ‘with metabolic syndrome but without fatty pancreas.’ Group comparisons showed significant differences in BMI (37.3 vs. 34.5 kg/m^2^, *p* = .022), waist circumference (115 vs. 109 cm, *p* = .037), LDL (2.6 vs. 1.9 mmol/L, *p* = .041), GGT (49 vs. 25 IU/L, *p* <.001), pancreatic T2* (36.46 vs. 41.78 ms, *p* = .009), and liver T2* (18.6 vs. 21.5 ms, *p* = .013) between the group ‘with both metabolic syndrome and fatty pancreas’ and that ‘with metabolic syndrome but without fatty pancreas’, respectively (Table 3).

**TABLE 3:** Clinical and laboratory characteristics of subjects with metabolic syndrome and with and without fatty pancreas.

Characteristics	With metabolic syndrome but without fatty pancreas (*n* = 13)	With both metabolic syndrome and fatty pancreas (*n* = 13)	*p*‐value
Age (year)	48 (35–52)	43 (28–52)	.072
Male/female (%)	3/10 (23.1/76.9)	5/8 (38.5/61.5)	.673
Smoking yes/no, *n* (%)	3/10 (23.1/76.9)	2/11 (15.4/84.6)	1.000
Drinking yes/no, *n* (%)	2/3 (40/60)	4/22 (15.4/84.6)	.417
BMI (kg/m^2^)	34.5 (28.3–40.8)	37.3 (33.6–44.6)	**.022**
Waist circumference (cm)	109 (95–121)	115 (111–125)	**.037**
Systolic blood pressure (mm Hg)	126 (100–142)	130 (107–143)	.144
Diastolic blood pressure (mm Hg)	80 (67–138)	83 (63–103)	.837
Antihypertensive drugs, *n* (%)	8 (72.7)	4 (44.4)	.928
Total cholesterol (mmol/L)	3.65 (2.8–6.2)	4.5 (3.2–5.1)	.055
Triglycerides (mmol/L)	1.6 (0.9–3.1)	1.6 (0.8–5.0)	.410
HDL‐c (mmol/L)	1.1 (0.6–1.5)	1.2 (0.8–1.5)	.797
LDL‐c (mmol/L)	1.9 (1.2–4.1)	2.6 (1.2–3.1)	**.041**
Albumin (mmol/L)	39.5 (5.0–44.0)	39 (34–43)	.743
Lipid‐lowering drugs, *n* (%)	7 (63.6)	4 (44.4)	.947
HBA1c (%)	6.5 (5.5–8.4)	7.1 (5.8–11.7)	.150
Fasting plasma glucose (mmol/L)	6.5 (3.9–9.1)	6.6 (5.3–10.9)	.644
Plasma fasting insulin (mIU/L)	27.2 (17.1–36.7)	25.1 (12.8–77)	.412
HOMA‐IR	6.90 (4.22–14.35)	7.89 (4.35–29.81)	.758
HOMA‐B	177.9 (82.33–1320)	187.0 (52.7–616)	.412
Insulin resistance, *n* (%)	13 (100)	13 (100)	1.000
T2DM, *n* (%)	11 (84.6)	11 (84.6)	1.000
Antidiabetic drug, *n* (%)	9 (81.8)	7 (77.8)	.953
ALP (IU/L)	53 (32–161)	72 (50–95)	.055
ALT (IU/L)	43 (19–132)	44 (13–107)	.918
AST (IU/L)	35 (19–96)	29 (16–45)	.106
GGT (IU/L)	25 (2.8–53)	49 (31–65)	**<.001**
VAT (litres)	4.14 (3.04–6.67)	4.15 (3.06–5.88)	.193
SAT (litres)	23.29 (12.12–29.47)	26.06 (16.10–33.96)	.713
VAT/SAT ratio	0.18 (0.13–0.55)	0.16 (0.11–0.24)	.689
Pancreatic PDFF (%)	3.9 (2.7–5.8)	8.3 (6.4–20.9)	**<.001**
Pancreatic T2* (ms)	41.78 (34.93–52.39)	36.46 (24.33–44.31)	**.009**
Liver PDFF (%)	12.7 (6.6–28.7)	17.9 (9.8–32.5)	.060
Fatty liver, *n* (%)	13 (100)	13 (100)	1.000
Liver cT1 (ms)	905 (724–1062)	907 (780–995)	.918
Liver T2* (ms)	21.5 (14.6–29.3)	18.6 (12.0–22.4)	**.013**

*Note*: Mann–Whitney test done for continuous variables and Fischer exact test for categorical variables. Bold indicates statistically significant values *p* < 0.05.

Abbreviations: ALP, alkaline phosphatase; ALT, alanine aminotransferase; AST, aspartate transaminase; BMI, body mass index; cT1, iron corrected T1; GGT, gamma‐glutamyl transferase; HbA1c, glycosylated haemoglobin, HDL, high density lipoprotein; HOMA‐IR, homeostasis model assessment‐insulin resistance; HOMA‐B, homeostasis model assessment beta; LDL, low density lipoprotein; PDFF, proton density fat fraction; SAT, subcutaneous adipose tissue; T2*, relaxation time; VAT, visceral adipose tissue.

### 3.4 T2DM and fatty pancreas

Due to the persistent significant difference observed in GGT in those with fatty pancreas, and the close relationship that exists between T2DM and GGT, we further performed a sub analysis on those with diabetes to rule out the influence of T2DM on the observed outcomes. Findings showed that only GGT (49 vs. 24 IU/L, *p* <.001), liver T2* (18.6 vs. 21.3 ms, *p* = .037), and age (43 vs. 48 years, *p* = .042) were significantly different between those ‘with both diabetes and fatty pancreas’ and those ‘with diabetes but without fatty pancreas’ (Table 4). Lower T2* times indicate presence of elevated iron content (high magnetic susceptibility effects) thus, lower T2* times.

**TABLE 4:** Clinical and laboratory characteristics of subjects with T2DM and with or without fatty pancreas.

Characteristics	With diabetes but without fatty pancreas (*n* = 12)	With diabetes and fatty pancreas (*n* = 12)	*p*‐value
Age (year)	48 (44–52)	43 (28–52)	**.042**
Male/female (%)	3/9 (25/75)	4/8 (33.3/66.7)	.500
Smoking yes/no, *n* (%)	3/9 (25/75)	1/11 (8.3/91.7)	.590
Drinking yes/no, *n* (%)	3/4 (42.9/57.1)	3/21 (12.5/87.5)	.110
BMI (kg/m^2^)	35 (28.3–40.8)	38 (33.6–44.6)	.068
Waist circumference (cm)	111 (95–124)	116 (111–127)	.053
Systolic blood pressure (mm Hg)	126 (102–142)	129 (107–143)	.563
Diastolic blood pressure (mm Hg)	80 (67–98)	80 (63–103)	.977
Antihypertensive drugs, *n* (%)	8 (88.9)	4 (50)	.625
Total cholesterol (mmol/L)	3.5 (2.8–6.2)	4.2 (3.2–4.9)	**.025**
Triglycerides (mmol/L)	1.6 (0.9–3.1)	1.5 (0.8–5.0)	.401
HDL‐c (mmol/L)	1.1 (0.6–1.7)	1.2 (0.8–1.5)	.953
LDL‐c (mmol/L)	1.8 (1.2–3.8)	2.5 (1.2–3.1)	**.032**
Albumin (mmol/L)	40 (5–44)	40 (34–43)	1.000
Lipid‐lowering drugs, *n* (%)	6 (66.7)	5 (62.5)	.995
HBA1c (%)	6.7 (5.9–8.4)	7.0 (5.7–11.7)	.644
Fasting plasma glucose (mmol/L)	6.6 (3.9–9.1)	6.6 (5.1–10.9)	.644
Plasma fasting Insulin (mIU/L)	27.7 (17.1–36.7)	26.9 (13.4–77.0)	.902
HOMA‐IR	6.89 (4.58–13.35)	8.48 (4.35–29.81)	.758
HOMA‐B	138.49 (82.33–1320)	190.43 (52.70–616)	1.000
Insulin resistance, *n* (%)	12 (100)	12 (100)	1.000
T2DM, *n* (%)	12 (100)	12 (100)	1.000
Antidiabetic drug, *n* (%)	9 (75)	8 (66.7)	.999
ALP (IU/L)	57 (40–161)	65 (50–102)	.185
ALT (IU/L)	43.5 (18–132)	45 (13–107)	.908
AST (IU/L)	32 (19–96)	28 (16–45)	.296
GGT (IU/L)	24 (2.8–44)	49 (31–65)	**<.001**
VAT (litres)	4.14 (3.04–6.67)	4.55 (3.26–5.88)	.322
SAT (litres)	24.11 (12.12–29.47)	26.34 (16.10–33.96)	.160
VAT/SAT ratio	0.18 (0.13–0.55)	0.16 (0.14–0.24)	.772
Pancreatic PDFF (%)	3.96 (2.7–5.8)	8.95 (6.4–20.9)	**<.001**
Pancreatic T2* (ms)	41.51 (28.65–47.46)	36.89 (24.33–44.31)	.053
Liver PDFF (%)	12.8 (6.6–21.1)	20.4 (9.8–32.5)	.052
Fatty liver, *n* (%)	12 (100)	12 (100)	1.000
Liver cT1 (ms)	908 (724–1062)	910 (817–995)	.751
Liver T2* (ms)	21.3 (14.6–29.3)	18.6 (13.0–22.4)	**.037**

*Note*: Mann–Whitney test done for continuous variables and Fischer exact test for categorical variables. Bold indicates statistically significant values *p* < 0.05.

Abbreviations: ALP, alkaline phosphatase; ALT, alanine aminotransferase; AST, aspartate transaminase; BMI, body mass index; cT1, iron corrected T1; GGT, gamma‐glutamyl transferase; HbA1c, glycosylated haemoglobin, HDL, high density lipoprotein; HOMA‐IR, homeostasis model assessment‐insulin resistance; HOMA‐B, homeostasis model assessment beta; LDL, low density lipoprotein; PDFF, proton density fat fraction; SAT, subcutaneous adipose tissue; T2*, relaxation time; VAT, visceral adipose tissue. Significance, *p* <.05.

### 3.5 Relationship between fatty pancreas and GGT

A logistic regression analysis showed that abnormal GGT levels (95% CI 2.422–621.655, *p* = .010) and hypertension (95% CI 0.004–0.940, *p* = .045) were the only significant independent predictors of fatty pancreas after controlling for sex, diabetes, alcohol intake, dyslipidaemia, hyperglycaemia, and hypoalphalipoproteinemia (Table 5), bearing in mind that all subjects had both central and general obesity. GGT also showed a continuous association with fatty pancreas by an OR of 7.333 (95% CI 1.467–36.664), while its diagnostic accuracy to determine fatty pancreas (AUC) was 0.849 (95% CI 0.693–1.000, *p* = .003) as shown in Table 6.

**TABLE 5:** Logistic regression analysis of fatty pancreas.

Variables in the equation	*B*	S.E.	Wald	*p*‐value	Exp (*B*)	95% confidence interval for EXP (*B*)	
						Lower	Upper
Sex	2.183	1.313	2.766	.096	8.877	0.677	116.329
Abnormal GGT levels	3.658	1.415	6.682	**.010**	38.802	2.422	621.655
Alcohol intake	−0.834	1.408	0.351	.554	0.434	0.027	6.864
Dyslipidaemia	0.760	1.279	0.353	.552	2.138	0.174	26.244
Diabetes mellitus	−0.962	1.967	0.239	.625	0.382	0.008	18.061
Hyperglycaemia	0.355	1.702	0.044	.835	1.427	0.051	40.061
Hypertension	−2.739	1.366	4.020	**.045**	0.065	0.004	0.940
Hypoalphalipoproteinemia	0.711	1.424	0.249	.618	2.036	0.125	33.215
Constant	−0.171	1.795	0.009	.924	0.843	‐	‐

*Note*: Bold indicates statistically significant values *p* < 0.05.

Abbreviation: GGT, gamma‐glutamyl transferase.

**TABLE 6:** Diagnostic accuracy of liver enzymes, abdominal adiposity, smoking, and drinking in determining fatty pancreas.

Variables	AUROC	Std. Error	*p*‐value	95% confidence interval	
				Lower bound	Upper bound
ALP	0.663	0.117	.165	0.434	0.892
AST	0.260	0.102	.041	0.059	0.460
GGT	**0.849**	0.080	**.003**	0.693	1.000
ALT	0.449	0.118	.663	0.218	0.680
VAT	0.622	0.114	.301	0.398	0.846
SAT	0.686	0.110	.115	0.470	0.901
BMI	0.673	0.113	.142	0.452	0.895
WC	0.728	0.120	.053	0.528	0.928
Smoking	0.503	0.118	.978	0.272	0.734
Drinking	0.506	0.118	.957	0.275	0.737

*Note*: Bold indicates statistically significant values *p* < 0.05.

Abbreviations: ALP, alkaline phosphatase; ALT, alanine aminotransferase; AST, aspartate transaminase; BMI, body mass index; GGT, gamma‐glutamyl transferase; SAT, subcutaneous adipose tissue; VAT, visceral adipose tissue; WC, waist circumference.

### 3.6 Fatty pancreas, GGT, and other covariates

Since certain medications, such as non‐steroidal anti‐inflammatory drugs, lipid‐lowering medications, antibiotics, histamine receptor blockers, antifungal agents, antidepressants, hormones, and ursodeoxycholic acid are known to increase GGT levels, we determined the impact of these medications. The results showed that they were 11 subjects with fatty pancreas and on medication known to increase GGT levels while they were 12 subjects without fatty pancreas but on medication known to increase GGT levels, *p* = .345. In terms of GGT levels between the two groups, it was shown that GGT levels were significantly higher in subjects with fatty pancreas who were on these medications compared to those without fatty pancreas on the same medications, GGT level of 36 (30–65 IU/L) versus 23.5 (2.8–53.0 IU/L), *p* = .004, respectively.

Similarly, since COVID‐19 vaccination has been shown to increase GGT levels, we conducted a sub‐analysis to determine its impact. We compared subjects who received the COVID‐19 vaccine and had fatty pancreas (*n* = 5) to those who received the vaccine but did not have fatty pancreas (*n* = 8), *p* = 1.000. The results indicated that GGT levels were significantly higher in subjects with fatty pancreas who were vaccinated compared to those without fatty pancreas who were also vaccinated, GGT level = 42 (32–51 IU/L) versus 24.5 (15–44 IU/L), *p* = 0.019, respectively.

Likewise, hypoalphalipoproteinemia has been shown to increase GGT levels. We thus compared subjects with hypoalphalipoproteinemia and had fatty pancreas (*n* = 9) to those with hypoalphalipoproteinemia but did not have fatty pancreas (*n* = 8), *p* = 1.000. The results indicated that GGT levels were significantly higher in subjects with fatty pancreas and with hypoalphalipoproteinemia compared to those without fatty pancreas but had hypoalphalipoproteinemia, GGT level = 36 (30–65 IU/L) versus 22.5 (2.8–32 IU/L), *p* = .004, respectively.

In this cohort, 6 subjects (19%) consumed alcohol within acceptable limits (<30 g/day for men and < 20 g/day for women). Among them, 3 (50%) had fatty pancreas with GGT levels of 32 (30–65 IU/L), and 3 (50%) did not have fatty pancreas with GGT levels of 44 (15–53 IU/L), *p* = .827. Additionally, 4 individuals (13%) were smokers. Of these, 3 (75%) had fatty pancreas with GGT levels of 51 (30–59 IU/L), and 1 (25%) did not have fatty pancreas with a GGT level of 2.5 IU/L, *p* = .180. All the above analyses which accounted for potential confounding factors on GGT levels, suggest that these factors did not significantly influence the observed outcomes.

### 3.7 Relationship between fat and iron

Pearson correlations showed that pancreatic PDFF was negatively associated with pancreatic T2* (*r* = −0.520, *p* = .005) and liver PDFF was equally negatively associated with liver T2* (*r* = −0.518, *p* = .002). Suggesting that the higher the fat content, the higher the iron content (as relaxation rate R2* is equal to the inverse of T2*).

## 4 DISCUSSION

In this study, we report for the first time the association of serum GGT with fatty pancreas in patients with obesity and with concurrent insulin resistance and MASLD without a history of pancreatitis. Several confounding factors have a great influence on serum GGT levels, such as excessive intake of alcohol, smoking, sex, pancreatitis, medication, COVID‐19 infection/vaccination, hypoalphalipoproteinemia, and liver injury, and were thus accounted for. One of the significant findings is that serum GGT was significantly elevated in patients with fatty pancreas independent of metabolic syndrome, diabetes, MASH, on medication known to increase GGT levels, hypoalphalipoproteinemia, and COVID‐19 vaccination/infection.

This study showed that elevated serum GGT levels were present in those with fatty pancreas irrespective of whether they had metabolic syndrome, diabetes, on medication known to increase GGT levels, or had COVID‐19 vaccination, similar to the findings from Maggio et al.^33^ It was further shown that abnormal GGT levels and hypertension were independent predictors of fatty pancreas. The risk (odd ratio) of fatty pancreas was shown to be 7.3 times higher when serum GGT was elevated. Moreover, it was shown that GGT had an AUROC of 0.849 in determining fatty pancreas. The possible explanation for this close relationship between fatty pancreas and GGT could be obtained from the functions of GGT. GGT among other structures is also present in the pancreatic cell membranes^8^ and helps in the metabolism of glutathione (an antioxidant), and leukotriene (stimulates inflammation),^34^ and facilitates the transfer of amino acids, peptides, and water across the cell membranes to preserve the intracellular homeostasis from oxidative stress.^9^, ^35^ The fatty infiltrations in the pancreas may cause cellular oxidative stress, mainly due to increased very low‐density lipoprotein (a finding consistent with our study) mediated transport of free fat acids, and changes in various adipokines.^36^ Due to this oxidative stress experienced in the pancreatic cells, more GGT may be released to metabolise glutathione and consequently protect the cellular integrity. Thus, the elevation in serum GGT may be indicative of increased oxidative stress experienced in the pancreatic cells including the beta‐cells. In conformity with our postulation, Corti et al.^37^ showed that serum GGT elevation reflects inflammation‐related oxidative stress.

Similarly, we showed that hypertension was closely related to fatty pancreas, consistent with previous studies.^26^, ^38^, ^39^ Persistent hypertension has been shown to increase oxidative stress on the pancreas.^40^ Oxidative stress is a key factor in pancreatic inflammation, which is often preceded by fatty infiltration (fatty pancreas). This may explain why both elevated GGT and hypertension are independently associated with fatty pancreas. Elevated serum GGT could serve as an early marker for oxidative stress related to intracellular disturbances in the pancreas, beyond liver injury. Indeed, elevated serum GGT has been linked to cardiovascular disease, stroke, dementia, diabetes, metabolic syndrome, cancer, and abnormal bone metabolism,^10^, ^41^ all of which involve structures containing GGT in their cell membranes.

Pancreatic T2* times were consistently shorter in those with fatty pancreas than in those without, indicating the presence of elevated iron content in those with fatty pancreas. A relationship that was also observed between PDFF and T2* in the liver. This outcome is similar with previous studies where iron deposition was associated with increased pancreatic fat^42^ or hepatic fat.^43^ Iron in the abdomen is predominantly deposited in the liver and to a lesser extent in the pancreas, especially in the beta‐cells. Iron is critical in the fuel oxidation and electron transport and its entry into the cells facilitates glucose and ethanol metabolism.^44^ However, iron has the potential to cause oxidative damage if it is not well‐regulated. It interacts with oxygen creating a toxic compound that can generate reactive oxygen species, and consequently cause damage to the deoxyribonucleic acid, phospholipids, and proteins.^45^

In view of the role that iron plays in the secretion of insulin by the pancreatic beta‐cells and how it increases with increase in fat deposition, as well as the close relationship that exists between the pancreas and the liver mediated in part through the regulation of glucose and lipid metabolism, we thus postulate that there could be liver‐pancreas crosstalk beyond the adipose tissue‐liver crosstalk to result in insulin resistance/beta‐cells dysfunction mediated by fatty infiltrations, inflammation, and iron deposition. Altogether, the effects of iron and fatty infiltrations associated with inflammation may induce oxidative stress in the pancreatic beta‐cells leading to the increased release of GGT.

Interestingly, waist circumference and BMI, simple anthropometric indexes consistently remained high in those with fatty pancreas regardless of whether the subjects had metabolic syndrome or diabetes. Contrastingly, VAT was not significantly different between groups, an outcome similar to our previous study in obese adolescents with MASLD.^5^ With regards to SAT, the significant difference observed between those with and without fatty pancreas was attenuated when analysed in those with metabolic syndrome and diabetes. Given that our study cohort had 74% and 57% of subjects on antidiabetic and lipid‐lowering drugs respectively, the non‐significant difference in VAT between those with and without fatty pancreas could have been due to the influence of these medications as they are known to alter body adiposity.^46^ Unlike our findings, other studies have shown that fatty pancreas is associated with increased VAT mass^33^, ^47^ albeit in study populations who were not on these medications, and not all had MASLD. Nevertheless, these outcomes suggest that although waist circumference and BMI cannot discriminate between VAT/SAT, and muscle/fat, respectively, their increase indicates the genesis of metabolic aberrations including fatty liver, fatty pancreas, and insulin resistance. Therefore, elevated serum GGT levels in individuals with general obesity in a clinical setting might suggest the need for a more detailed assessment, which may include radiological evaluation, of both the liver and the pancreas. This could be vital because fatty pancreas is associated with deleterious outcomes such as diabetes^7^ and pancreatic cancer.^48^

This study has several limitations that need to be acknowledged. First, this was a single‐centre cross‐sectional study with a relatively small sample size. Second, we could not establish the cause‐and‐effect relationship between GGT and fatty pancreas. Third, the study cohort had obesity, insulin resistance, and MASLD, thus may not be representative of the general population. Fourth, HOMA‐B and HOMA‐IR indexes were used, which are less accurate in reflecting beta‐cell function and insulin resistance in comparison with the glucose challenge test and hyperglycaemic clamp method, respectively. Fifth, although we used iron‐corrected T1 at a cut‐off point of 925 ms to rule in MASH as was shown in previous studies and validated against histology.^31^, ^32^ Liver biopsy remains the gold standard and therefore like other non‐invasive tests, there could be a possibility of missed MASH diagnosis using this non‐invasive biomarker. Furthermore, the absence of oxidative stress markers, MR elastography, and/or biopsy to determine the presence of fibrosis are limitations that can confound the results. Finally, we did not investigate the genetic makeup inherent differences in GGT in our Chinese cohort and we did not have a control group. Thus, our results should be interpreted with caution.

In conclusion, elevation in serum GGT might be a potential marker to identify fatty pancreas. Early diagnosis of fatty pancreas could provide an opportunity for further patient‐detailed investigations as well as an opportunity for clinicians to advise patients on the risks linked to this condition. It further gives clinicians a chance to institute early personalised interventions such as lifestyle modifications to improve the metabolic profile. Future studies are warranted involving large sample sizes in those with fatty pancreas only (without MASLD) to validate these findings.

## AUTHOR CONTRIBUTIONS

**CC**: conceptualization, methodology, investigation, formal analysis, writing original draft, writing—review and editing. **KHL**: investigation, writing—review and editing. **ES**: writing—review and editing. **TCFY**: statistical analysis, review, and editing. **VW**: writing—review and editing. **WC**: supervision, methodology, project administration, investigation, writing—review and editing, funding acquisition. All authors were involved in writing the paper and had final approval of the submitted and published versions.

## FUNDING INFORMATION

This project was funded in part by the Chinese University of Hong Kong, Direct Grant for Research (Ref. no: 2019.015).

## CONFLICT OF INTEREST STATEMENT

The authors declare no conflict of interest.

## Supporting information


**Supplementary Table S1.** Showing the medication subjects were receiving 6 months from MRI examination date.
