# Supplementary material for: Gamma‐glutamyl transferase: A potential biomarker for pancreas steatosis in patients with concurrent obesity, insulin resistance and metabolic dysfunction‐associated steatotic liver disease
Source: Clin Obes. 2024 Oct 22;15(1):e12712. doi: 10.1111/cob.12712 (PMC11706757; doi:10.1111/cob.12712)
Supplement: Supplementary file 1 — Supplementary Table S1. Showing the medication subjects were receiving 6 months from MRI examination date. [file COB-15-e12712-s001.pdf]

Gamma-glutamyl transferase: a potential biomarker for pancreas steatosis in patients with concurrent obesity, insulin resistance and metabolic dysfunction-associated steatotic liver disease

Chileka Chiyanika, BSc, PgD, MSc, PhD[1,2], Elizabeth Shumbayawonda, BSc, MSc, PhD[6], Michele Pansini, MD[6,7,8], Kin Hung Liu, BSc, MPhil, PhD[2], Terry Cheuk-Fung Yip, BSc, MPhil, PhD[4,5], Vincent Wai-Sun Wong, MBChB, FHKCP, FHKAM, MD, FRCP (Edin), FRCP (Lond) [3,4,5], Winnie Chiu Wing Chu, MBChB, FRCR, FHKCR, FHKAM, MD [2]

1. Department of Health Technology and Informatics, The Hong Kong Polytechnic University, Hong Kong, China.
2. Department of Imaging and Interventional Radiology, Prince of Wales Hospital, The Chinese University of Hong Kong, Hong Kong, China.
3. Institute of Digestive Disease, The Chinese University of Hong Kong, Hong Kong, China.
4. Medical Data Analytic Centre, The Chinese University of Hong Kong, Hong Kong, China.
5. Department of Medicine and Therapeutics, The Chinese University of Hong Kong, Hong Kong, China.
6. Perspectum Diagnostic limited, Oxford, England.
7. Clinica Di Radiologia EOC, Istituto Di Imaging Della Svizzera Italiana (IIMSI), Ente Ospedaliero Cantonale, Via Tesserete 46, 6900, Lugano, Switzerland
8. John Radcliffe Hospital, Oxford University Hospitals NHS Foundation Trust, OX3 0AG, Oxford, UK

CORRESPONDING AUTHOR ADDRESS:

Professor Winnie Chiu Wing Chu
Department of Imaging and Interventional Radiology,
Room 27026,
Ground Floor, Treatment Block and Children Wards,
Prince of Wales Hospital, Shatin, Hong Kong SAR, China.
Tel: (852) 3505 2299
Fax: (852) 2636 0012
Email: winniechu@cuhk.edu.hk

CONTACT INFORMATION

- Chileka Chiyanika: Hong Kong Polytechnic University, Room Y938, 11 Yuk Choi Road, Hung Hom, Hong Kong SAR, China. Email: chileka.chiyanika@polyu.edu.hk
- Elizabeth Shumbayawonda: Perspectum Diagnostics, Gemini One, 5520 John Smith Drive, Oxford, OX4 2LL, United Kingdom. Email: elizabeth.shumbayawonda@perspectum.com
- Michela Pansini: Perspectum Diagnostics, Gemini One, 5520 John Smith Drive, Oxford, OX4 2LL, United Kingdom. Email: michele.pansini@perspectum.com
- Kin Hung Liu: The Chinese University of Hong Kong, Prince of Wales Hospital, Department of Imaging and Interventional Radiology, G/F, Old Block, Hong Kong. Email: lkh605@ha.org.hk
- Terry Cheuk-Fung Yip: The Chinese University of Hong Kong, Room 74046, 5/F, Lui Che Woo Clinical Sciences Building, Prince of Wales Hospital, Shatin, Hong Kong. Email: tcfyip@cuhk.edu.hk
- Vincent Wai-Sun Wong: The Chinese University of Hong Kong, Department of Medicine and Therapeutics, 9/F, Prince of Wales Hospital, Shatin, Hong Kong. Email: wongv@mect.cuhk.edu.hk

Supplementary Table (S1). Showing the medication subjects were receiving 6 months from MRI examination date

ID	Name of medicine/drug received in the last 6 months prior to MRI	Use of at least one drugs known to affect GGT levels (0=no, 1=yes)
1	metformin, amlodipine, simvastatin	1
2	metformin, amlodipine, simvastatin	1
3	indomethacin, amoxycilin, bonjela, thymol gargle compound, Hypromellose, prednisolone, metformin, amlodipine	1
4	chloramphenicol, amitriptyline, Augmentin, metformin, amlodipine, simvastatin	1
5	Dexamethasone, metformin, amlodipine, simvastatin	1
6	metformin, amlodipine, simvastatin	1
7	Hydroxzine, Dapagliflozin, stieprox, betahistine, famodine, Aqueous	1
8	prednisolone, metformin, amlodipine	0
9	Hypromellose, metformin	0
10	Dexamethasone, Augmentin, chloramphenicol, amlodipine, hypromellose, metformin	1
11	glipizide, Janumet, famotidine	1
12	thymol gargle compound, mefenamic acid, bonjela, budesomide, clotrimazole, cetrizine, diclofenac, famotidine, losartan, montelukast	1
13	metformin, betahistine, metoclopramide, tramadol, norethisterone, bromhexine, chlorpheniramine, cocillana, dioctahedral smectite, ammonia and ipecacuanha, norvasc	1
14	acyclovir, augmentin, calamine, metformin, amlodipine, simvastatin	1
15	amlodipine, dexamethazone	0
16	insulin, ibuprofen, hypromellose, metformin	1
17	ibuprofen, dexamethazone	1
18	Dapagliflozin, insulin, amlodipine, pantoprazole	0
19	Dexamethasone, ampicillin, cloxacillin, ceftriaxone, metformin, amlodipine, simvastatin	1
20	metformin, simvastatin, famotidine	1
21	Dapagliflozin, metformin, dexamethazine	0
22	Dapagliflozin, dexamethasone, metformin, simvastatin	1
23	Dapagliflozin, dexamethasone	0
24	Dapagliflozin, metamucil, amlodipine	0
25	Dapagliflozin, dexamethazone	1
26	Dapagliflozin, metformin, amlodipine	0
27	Dapagliflozin, ammoniam and ipecacuanha, Dequalinium, loratadine, simvastatin	1
28	Dapagliflozin, melatonin	0
29	Dapagliflozin, amlodipine, pioglitazone, simvastatin, glimepiride, ofloxacin, augmentin	1
30	Dapagliflozin, dioctahedral smectite, hyoscine, bromocriptine, diclofenac, medroxyprogesterone, methyl salicylate compound	1
31	Dapagliflozin, amoxycilin, clarithisterone, simvastatin, norethisterone, amlodipine	1
